# Impact of COVID-19 on Key Performance Indicators of the National Hip Fracture Database and the Management of Hip Fracture Patients

**DOI:** 10.7759/cureus.20575

**Published:** 2021-12-21

**Authors:** Jennifer L Waterman, Ullas Jayaraju, Joshua K Nadimi, David Morgan

**Affiliations:** 1 Surgery, Royal Glamorgan Hospital, Llantrisant, GBR; 2 Trauma and Orthopaedics, Royal Glamorgan Hospital, Llantrisant, GBR

**Keywords:** theatre efficiency, covid-19, 30 day mortality, neck of femur fractures, trauma

## Abstract

Background: A hospital's performance regarding the management of hip fractures is based on six key performance indicators (KPIs) which are recorded onto the National Hip Fracture Database (NHFD). The aim of this study was to assess the overall impact of coronavirus disease 2019 (COVID-19) on the management and outcomes of hip fracture patients against a similar period in 2019 by utilizing the KPIs.

Method: Retrospective data collection of hip fracture patients during a six-week (pre-COVID) period in 2019 and a six-week (COVID-19) period in a single orthopedic unit. The following parameters were compared; patient age, time to theater, surgeon operating time, total time in the operating room, time from ward to recovery, time from hospital presentation to theater, and total time from presentation to hospital discharge.

Results: Some 38 patients in the pre-COVID-19 period vs. 27 patients with hip fractures in the COVID-19 period were included in the study. Time from diagnosis to theater and surgeon operating time were similar in both groups. The mean length of stay was 9.3 days vs. a mean of 31.34 days (p = 0.0004) in the COVID-19 and pre-COVID-19 groups respectively. A 30-day mortality was 22.2% (n = 6) in the COVID-19 group vs. 5.3% (n = 2) in the pre-COVID-19 group.

Conclusion: Our study demonstrates that the combination of surgical stress and COVID-19 leads to higher mortality rates. Our hospital’s structural reorganization during the pandemic has shown progress in achieving important KPIs and improved short-term outcomes for hip fracture and trauma patients.

## Introduction

Annually there are around 75,000 hip fractures managed within the National Health Service (NHS) [1]. Guidelines for the management of these fractures are provided by the British Orthopaedic Association (BOA) and the National Institute of Health and Clinical Excellence (NICE) [1-2]. A hospital's performance is based on six key performance indicators (KPIs) which are recorded onto the National Hip Fracture Database (NHFD), evaluated, and presented on an annual basis to compare performance throughout the country [3].

The coronavirus disease 2019 (COVID-19) pandemic has delivered significant challenges to the orthopedic community, which have resulted in huge changes in the delivery of services. Nonetheless, the management of hip fractures, ‘remains urgent and a surgical priority,’ as described by the BOA [4].

Pre-COVID-19, our department dedicated a one-session operating list daily (morning or afternoon). In response to the COVID-19 pandemic, our hospital reorganized its Trauma and Orthopedic (T&O) services, by canceling elective operations and an all-day trauma list was initiated [5].

Patients suspected of contracting COVID-19 were placed in cohort areas until their COVID-19 swab results were confirmed. They were then transferred to either a COVID-19 ward or a clean (negative) ward. A COVID-19 theater was created where positive or suspected patients underwent surgery. Public Health England has produced guidance on the extra-precautionary measures and personal protective equipment (PPE) required during aerosol-generating procedures. Furthermore, an editorial from The Bone and Joint Research Journal advised avoiding diathermy and limiting the use of power tools during fracture fixation and instrumentation [6-7]. These intra-operative precautions combined with guidance on airway management in theater are predicted to cause a detrimental effect on the fluency of the theater list [8]. The anticipated effect of additional PPE in combination with the stringent precautions taken in the theater when conducting aerosol-generating procedures is likely to increase the patient's total time in the theater.

The aim of this study was to assess the overall impact of COVID-19 on the management and outcomes of hip fracture patients in our hospital against a similar period in 2019 by utilizing the KPIs from the NHFD. We hypothesize that time to theater and discharge time/length of stay in hospital will be overall faster, however, mortality will increase during the COVID-19 pandemic when compared to the same time period in 2019.

## Materials and methods

Retrospective data collection was performed on all hip fracture patients between 23/03/2019 and 05/05/2019 (pre-COVID-19 cohort) and compared with hip fracture patients presenting between 23/03/2020 and 05/05/2020 (COVID-19 pandemic cohort) from a single orthopedic unit.

All patients over the age of 18 with a hip fracture who underwent operative management during the period stated were included in the study. This covered both intracapsular and extracapsular neck of femur (NOF) fractures. The following parameters were analyzed/compared; patient age, time to theater, surgeon operating time, total time in the operating room, time from ward to recovery, time from hospital presentation to theater, and total time from presentation to hospital discharge.

Hospital discharge was defined as any patient leaving the acute hospital to go back to their original, new residence or rehabilitation hospital. This information was obtained via our online password-secure theater intranet system.

A 30-day postoperative patient mortality was also reviewed by utilizing our all Wales online intranet system in addition to causes of death as recorded on death certificates.

Data from both periods were analyzed and compared to assess the impact of the COVID-19 pandemic on our management and outcomes of hip fractures. Unpaired two-tail t-testing for parametric data was performed on both cohorts to assess the significance of observed differences for each selected time parameter.

## Results

A total of 38 patients with hip fractures presented to our unit in the pre-COVID-19 period (23/03/2019 to 05/05/2019) compared to 27 patients with hip fractures in the COVID-19 pandemic period (23/03/2020 to 05/05/2020). The mean age for these groups were 80.31 years (range 62-94 years) and 80.30 years (range 50-97 years) for the pre-COVID and COVID groups, respectively. There were 15 male and 23 female patients in the pre-COVID group and 9 male and 18 female in the COVID group.

There were 29 intracapsular NOF fractures and 10 extracapsular femoral fractures sustained in the pre-COVID-19 cohort. One patient from the pre-COVID-19 sample sustained bilateral NOF fractures. Operations performed were 22 unipolar hip hemiarthroplasty, five cannulated hip screw fixations, two total hip replacements (THR), eight dynamic hip screw (DHS) fixations, and two cephalomedullary femoral nail fixations.

Within the COVID-19 cohort, there were 18 intracapsular NOF fractures and 10 extracapsular femoral fractures. One patient from the COVID-19 sample group sustained bilateral NOF fractures. Procedures performed included eight DHS fixations, two cannulated hip screw fixations, 15 unipolar hemiarthroplasties, one THR, and two cephalomedullary femoral nails.

One patient from the COVID-19 sample group sustained bilateral NOF fractures which required both a cephalomedullary femoral nail and DHS fixation during one operative case and one general anesthetic. One patient from the pre-COVID-19 sample also sustained bilateral NOF fractures requiring two unipolar hip hemiarthroplasty procedures during the same operative case/anesthetic.

Mean values and statistical significance, p-values, for specific parameters studied in this project for both pre-COVID-19 and COVID-19 can be found in Table 1.

**Table 1:** Mean values and statistical significance, p-values, for specific parameters studied in this project both pre-COVID-19 and COVID-19.

Time period		Pre-COVID-19 (n=38)	COVID-19 (n=27)		p (T<=t) two-tail
Time from diagnosis to theater (h)	Mean	28.087 (5.467-93.42)	24.548 (6.42-50.65)		0.342
	Variance	265.046	179.839		
Call for patient to anesthetic room (min)	Mean	19.763 (11-36)	24.074 (11-41)		0.025
	Variance	28.131	72.994		
Surgeon operating time (min)	Mean	71.500 (36-179)	73.222 (34-227)		0.839
	Variance	757.446	1383.79		
Total time in theater (TTT) (min)	Mean	97.210 (54-209)	116.259 (53-298)		0.074
	Variance	897.143	2283.814		
Time from ward to recovery (min)	Mean	157.315 (105-258)	152 (98-315)		0.611
	Variance	1125.141	2103.692		
Time from presentation to discharge/Length of stay (days)	Mean	31.343 (6-125)	9.333 (2-26)		0.0004
	Variance	1092.349	41.533		

Time from diagnosis to theater in the COVID-19 group was 24.5 h compared to the 28.09 h in the pre-COVID-19 group. In the pre-COVID-19 group, 28.5% (n=11) underwent an operation more than 36 h after diagnosis and the COVID-19 hip fracture cohort was found to have eight patients (29.6%) undergoing an operation after the 36 h period.

Surgeon operating time pre-COVID was a mean of 71.5 and 73.2 min during COVID-19.

Analysis of total time from hospital presentation to discharge demonstrated a mean of 31.34 days (pre-COVID) vs. 9.3 days (COVID), showing a 71% decrease in overall hospital stay; this was statistically highly significant (p = 0.0004). Surgeon operating times for pre-COVID-19 and COVID-19 groups were comparable at 72 min (range 36-179 min) and 73 min (range 34-227 min) (p = 0.839).

Pre-COVID-19 mean theater time was 97.2 min vs 116.3 min during COVID, demonstrating a 19.6% increase in total theater during COVID, not statistically significant between the two groups (p = 0.074).

A higher 30-day mortality rate was observed in the COVID-19 group with six out of 27 patients (22.2%) when compared to the pre-COVID-19 group, with two out of 38 patients (5.3%) dying within the 30-day period. The cause of death is shown in Table 2.

**Table 2:** Mortality in COVID-19 cohort. Breakdown of patient ASA, medical history, and COVID-19 status. ASA, American Society of Anaesthesiologists; AF, atrial fibrillation; CKD, chronic kidney disease; MI, myocardial infarction; HTN, hypertension; OSA, obstructive sleep apnea; PE, pulmonary embolism

Patient	Age	ASA score	Procedure/Operation	Comorbidities	COVID result	Day of COVID swab post presentation	Cause of death
A	76	4	Dynamic hip screw fixation	Parkinson’s disease	Not tested	n/a	PE
B	88	4	Unipolar hemiarthroplasty	AF, CKD, Bladder Ca, Diabetes, MI	Positive	4	COVID-19
C	91	3	Unipolar hemiarthroplasty	CKD, HTN, Breast Ca + Mastectomy	Positive	10	COVID-19
D	93	3	Dynamic hip screw fixation	Rheumatic Fever, HTN	Positive	8	COVID-19
E	83	3	Cannulated hip screw fixation	Dementia, Bipolar, CKD	Positive	21	COVID-19
F	50	3	Dynamic hip screw fixation	Down syndrome, Budd-Chiari syndrome, OSA	Positive	4	COVID-19

## Discussion

As of June 2020, there were more than 40,000 COVID-19 related deaths in the UK [9]. Hip fracture patients are arguably the most vulnerable of T&O patients and this pandemic has heightened our need to manage these patients swiftly to minimize the risk of coronavirus to both patient and hospital staff, whilst maintaining optimum patient care [10].

The principal finding of our study showed patients in the COVID-19 group spent a shorter period of time in hospital prior to discharge. Enhanced recovery protocols are well established in orthopedic practice, with the aim of decreasing the patient length of stay [11]. With elective services canceled, multidisciplinary teams are able to better focus these services on the recovery and discharge of trauma patients. In our unit, the orthopedic physiotherapists, occupational therapists, and advanced nurse practitioners normally assigned to the management of elective patients were available to add extra resources to the trauma team. This has led to a more efficient patient pathway from admission to discharge.

The second KPI states that patients should undergo prompt surgery by the following day of after presentation with a hip fracture [12]. When comparing our two cohorts of patients, time to theater from diagnosis was faster during the COVID-19 period, with patients undergoing an operation at a mean time of 24.5 h. We believe this swifter movement of patients to theater is due to a combination of a reduction in the number of trauma patients, a more focused hip fracture pathway, and a dedicated all-day trauma theater list, specifically, introduced during the COVID-19 pandemic.

The NHFD Annual Report in 2019 found factors relating to patients not having prompt surgical intervention were classified as logistic and administrative reasons (awaiting space on a trauma list or that a list over-ran resulting in cancelation of a procedure) [13]. Our results indicate that a two-session (all day) trauma list allowed us to manage our hip fracture patients optimally by reducing wait-times and receiving surgical intervention faster. A larger retrospective study is required to further evaluate the sustainability of the all-day trauma list alongside elective operating lists.

Patients and theater staff spent longer total time to theatre (TTT) with the COVID-19 cohort. The reasons for an increase in TTT are multifold, including additional anesthetic time and surgeon operating time due to an increase in PPE and theater precautions [7]. During the pandemic, new protocols for airway management were introduced. Aerosol generating procedures such as anaesthetization and extubation of patients were performed in the operating room, thereby increasing overall TTT [14]. Prolonged surgeon operating time has been associated with increased risk of perioperative complications, however, our study showed that mean operating time was similar in both cohorts (71.5 min vs. 73.2 min) [15]. The comparable result in both cohorts is an important observation and shows pandemic-related changes, such as the additional PPE and theater precautions, have had a small effect on the surgeon’s ability to perform operations on hip fractures.

Another KPI reviewed by the NHFD is the return to normal residence [3]. Of our COVID-19 group, 44% (n = 12) returned home and 41% (n = 11) were discharged for further rehabilitation to community hospitals. Pre-COVID-19 82% of patients (n = 31) returned home. The NHFD Annual Report in 2019 found the mean acute hospital length of stay was 15.1 days [13]. Our pre-COVID-19 group was found to have a mean of 31.34 days length of stay in hospital. But the COVID-19 group had a substantially lower (statistically significant improvement, p = 0.004) time in hospital with a mean of 9.3 days length of stay. This reduced length of stay in hospital is essential in minimising transmission of COVID-19 to hip fracture patients and also reducing mortality [16-17].

A similar proportion of patients in the COVID-19 group returned home or were stepped down to a rehabilitation center but this was achieved at a faster rate than during the pre-COVID-19 period. This is likely due to the enhanced physiotherapy and occupational therapy pathways to ensure ongoing acute trauma and orthopedic bed availability to treat patients and reduce the risk and spread of COVID-19 to patients admitted into hospital. It is feasible to state that patients in the pre-COVID-19 group underwent a longer rehabilitation process in hospital to then be discharged home rather than be stepped down to a rehabilitation hospital due to lack of beds (from both trauma and elective patients undergoing rehab). Elective operations were canceled during the COVID-19 period and this would have had a positive impact on physiotherapy services as well as increased bed availability in the step down rehabilitation hospitals. This efficient discharge pathway has been critical in faster patient flow in and out of the hospital, during the COVID-19 period. We believe that this discharge system should be maintained throughout and beyond the COVID-19 period to reduce strain on acute hospital trauma beds.

The 30-day mortality rate is a well-established guide for assessing outcomes following NOF fractures [18]. The number of deaths during this 30-day period was found to be higher in our COVID-19 group vs. the pre-COVID-19 group. A study from New York found patients with hip fractures who tested positive for COVID-19 had increased mortality rate than those who tested negative (35.3% vs. 0.9%) between the period of February 1 and April 15 2020 [19]. A recent international multicenter study found the 30-day mortality in patients undergoing surgery with peri-operative COVID-19 was 23.8% [20]. The Intensive Care National Audit and Research Centre (ICNARC) report on COVID-19 in critical care found 68.1% of patients over the age of 70 died whilst in critical care with COVID-19 [21]. This is in keeping with our findings, of a mean age of 81 years for patients who died of COVID-19 following operation for NOF fracture. Research will need to continue to further understand the acute and long-term effects of COVID-19 and NOF fracture patients.

Limitations of our study include a retrospective review, with a small cohort over a short time frame. A further limitation is that pre-operative comorbidities were not compared in this study. However, our data include follow-up outcomes for 30-day mortality. This work is intended as an early review of the impact of COVID-19 on the management of hip fractures by evaluating against the NFHD KPIs.

## Conclusions

Our study demonstrates that the combination of surgical stress and COVID-19 leads to higher mortality rates. Our hospital’s structural reorganization during the pandemic to improve safety for hip fracture patients and trauma patients has shown improvements in important KPIs leading to more efficient and improved short-term outcomes. This is through ensuring an all-day trauma theater list, a senior surgeon present intra-operatively, and resources aimed at discharging patients and thereby reducing the overall hospital length of stay.
